# *In-vivo* RGB marking and multicolour single-cell tracking in the adult brain

**DOI:** 10.1038/srep07520

**Published:** 2014-12-22

**Authors:** Diego Gomez-Nicola, Kristoffer Riecken, Boris Fehse, V. Hugh Perry

**Affiliations:** 1 Centre for Biological Sciences, University of Southampton, Southampton, United Kingdom; 2 Research Department Cell and Gene Therapy, Clinic for Stem Cell Transplantation, University Medical Centre Hamburg-Eppendorf, Hamburg, Germany

## Abstract

In neuroscience it is a technical challenge to identify and follow the temporal and spatial distribution of cells as they differentiate. We hypothesised that RGB marking, the tagging of individual cells with unique hues resulting from simultaneous expression of the three basic colours red, green and blue, provides a convenient toolbox for the study of the CNS anatomy at the single-cell level. Using γ-retroviral and lentiviral vector sets we describe for the first time the *in-vivo* multicolour RGB marking of neurons in the adult brain. RGB marking also enabled us to track the spatial and temporal fate of neural stem cells in the adult brain. The application of different viral envelopes and promoters provided a useful approach to track the generation of neurons vs. glial cells at the neurogenic niche, allowing the identification of the prominent generation of new astrocytes to the striatum. Multicolour RGB marking could serve as a universal and reproducible method to study and manipulate the CNS at the single-cell level, in both health and disease.

The complex organisation of the central nervous system (CNS) requires sophisticated approaches to identify and modify the phenotype of individual cells in order to determine their function in the healthy and diseased brain. The field of neuroscience is rapidly expanding and adapting several molecular tools to achieve these goals. One very elegant approach is the Brainbow mouse, which uses the stochastic expression of fluorescent proteins with different colours in a cellular population, leading to a combinatorial expression of these proteins creating multiple colours^1^,^2^. It has allowed spectacular insights, highlighting the cellular complexity of the developing and adult brain. That approach, similar to its technical predecessors, the expression of GFP spectral variants^3^ and the MADM method (mosaic analysis with double markers)^4^, requires the transgenic modification of mice. Besides advantages of the use of transgenic mice, some disadvantages include limited cellular specificity of the fluorescent labelling, limited options for timing and spatial distribution of the labelling, restricted (immediate) availability for the broad scientific community, and the fact that even small modifications require time-consuming breeding programmes. The field of neuroscience has also benefited from the use of viral approaches for the study of the generation and fate of neural stem cells. The use of lentiviral^5^ or γ-retroviral^6^ vectors to drive the expression of fluorescent proteins, such as GFP, to investigate neurogenesis provided the basis for a set of studies focused on the generation, migration and differentiation of newly generated neurons in the subventricular zone or the dentate gyrus of the hippocampus. Although a recent upgrade of Brainbow technology was transferred to adeno-associated viral vectors^7^, customizable and inheritable single-cell colour-coding is still not possible for the study of brain anatomy and function.

An alternative approach that has offered valuable insights to the study of the developing brain is the use of multicolour labelling by electroporation of plasmids, namely the StarTrack^8^, MAGIC^9^ and CLoNe^10^ methods. However, these approaches are limited to the study of embryonic or early postnatal brain, without direct applicability to study the healthy and diseased adult brain. Taken together, existing methods have some limitations since they do not readily permit the investigator to perform single-cell analysis, or more precise temporal or spatially dynamic studies. A new method to perform single-cell analysis of neural stem cells and their progeny, together with the ability to manipulate gene functions and the flexibility to use it in any mouse model without transgenesis would serve as a solid base to further our understanding of neural stem cell physiology and the molecular regulation of neurogenesis in both health and disease.

Recently, we extended the use of fluorescent protein-based cell marking by applying the principle of RGB colour mixing^11^,^12^. The simultaneous, lentiviral-vector mediated expression of three genes encoding fluorescent proteins in the three basic colours, red, green and blue, results in multicolour labelling of different cell populations, to be used *in vitro* and *in vivo*^11^. This technique facilitates clonal analysis and cell tracking over long periods of time. Moreover, the use of lentiviral gene ontology (LeGO) vectors^13^,^14^ for RGB marking makes possible the concurrent investigation of gene functions in marked cells. LeGO vectors are designed according to the building-block principle, allowing the co-expression of (drug-selectable) fluorescent proteins and genes-of-interest and/or short-hairpin RNAs (shRNAs), providing a flexible and potent toolbox for the over-expression or down-regulation of potentially interacting genes^13^,^14^. Here we show that the application of the RGB-marking technique with LeGO vectors to the study of the CNS provides a valuable set of tools to perform long-term *in-vivo* single-cell analysis of glial or neuronal lineages or populations and to perform analysis of cell progenies, opening a new scenario for the study of CNS development and physiology. We report on the preparation of novel population-specific lentiviral and γ-retroviral vectors containing different promoters and the first *in-vivo* application of single-cell multicolour RGB marking to the study of mature neuronal populations and the temporal and spatial dynamics of neurogenesis at the subventricular zone and the dentate gyrus, providing the basis for a broadly applicable method to track and manipulate CNS cells.

## Results

### Design, preparation and characterisation of RGB lentiviral and γ-retroviral vectors

When we first published the technique of RGB marking^11^, we used LeGO vectors^14^ for the transfer of the three fluorescent proteins mCherry (red), Venus (green) and Cerulean (blue) under the control of the potent and ubiquitous SFFV promoter^15^,^16^ (SFFV-LV). To analyse the impact of the selected promoter on the outcome of cell marking in the brain, we cloned a new set of lentiviral RGB vectors (CMV-LV) containing the widely used immediate early promoter of the human cytomegalovirus (CMV), known for robust expression and high titres when used in lentiviral vectors^17^.

To broaden the applicability of RGB marking in the brain even further, we tried to make use of an often-stated “disadvantage” of MLV-derived γ-retroviral vectors, the inability to transduce non-dividing cells^18^,^19^. This could be easily turned into an advantage, if proliferating (stem) cells are to be marked exclusively. We therefore cloned a set of three γ-retroviral vectors (SFFV-RV) based on RSF91.GFP.pre* (18), expressing the three fluorescent proteins for RGB marking (red, green and blue) under the control of the SFFV promoter. All vectors are displayed in Supplementary Figure 1.

**Figure 1:**
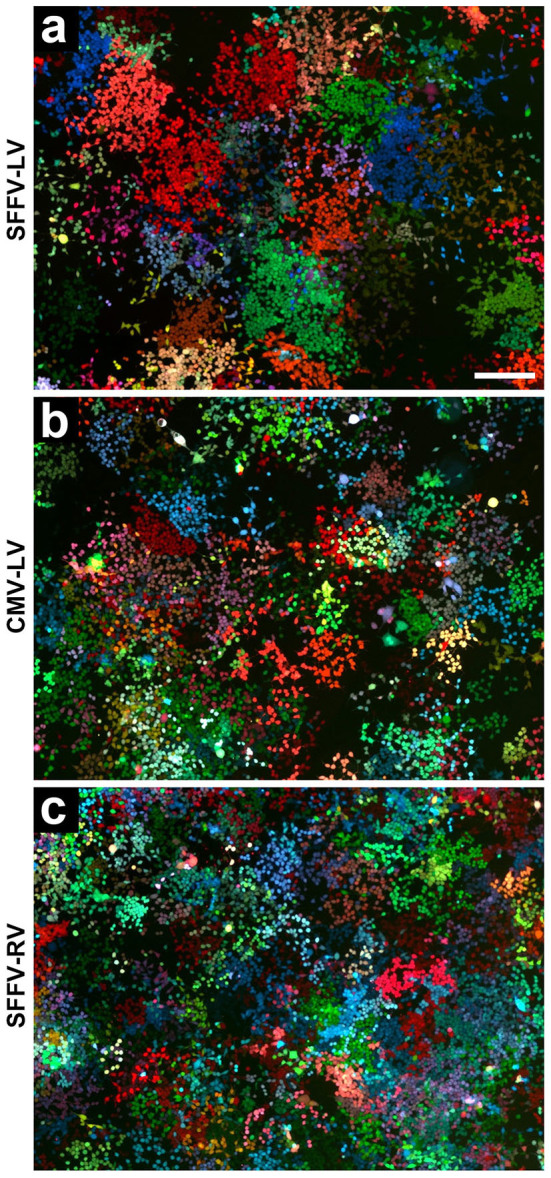
RGB marking of cultured cells with lentiviral and γ-retroviral vectors. RGB marking of HEK-293T cells, transduced with (A) VSVG pseudotyped SFFV-promoter containing lentiviral vectors (VSVG-SFFV-LV), (B) VSVG pseudotyped CMV-promoter containing lentiviral vectors (VSVG-CMV-LV), or (C) VSVG pseudotyped SFFV-promoter containing γ-retroviral vectors (VSVG-SFFV-RV). Cells in (A) are shown 6 days after plating, cells in (B–C) are shown 5 days after plating, indicating the correlation between cell growth and size of homogenously coloured cell clusters. (A–C) Fluorescence is shown in red (mCherry), green (Venus) and blue (Cerulean). All images were taken from live cells in an Olympus IX81 inverted microscope by stitching images taken with a colour camera and a 10x objective. Scale bar: A–C, 250 µm (in A).

The VSVG-pseudotyped SFFV-LV (VSVG-SFFV-LV) provided RGB marking of 293T cells (Figure 1a) as previously described^11^,^12^. The use of the CMV promoter (VSVG-CMV-LV) instead of the SFFV promoter also allowed reproducible and stable RGB marking (Figure 1b). RGB marking with VSVG-pseudotyped γ-retroviral vectors (VSVG-SFFV-RV) was as effective as with the lentiviral vectors (Figure 1c). Comparative FACS analyses of the three sets of RGB vectors are given in the supplementary data (Supplementary Figure 2).

We hypothesized that these new lentiviral and γ-retroviral vectors provide a toolbox for the multicolour RGB marking and tracing of different, post-mitotic as well as proliferating cell populations in the CNS.

### RGB allows for marking of neuronal populations at the single-cell level

**Figure 2:**
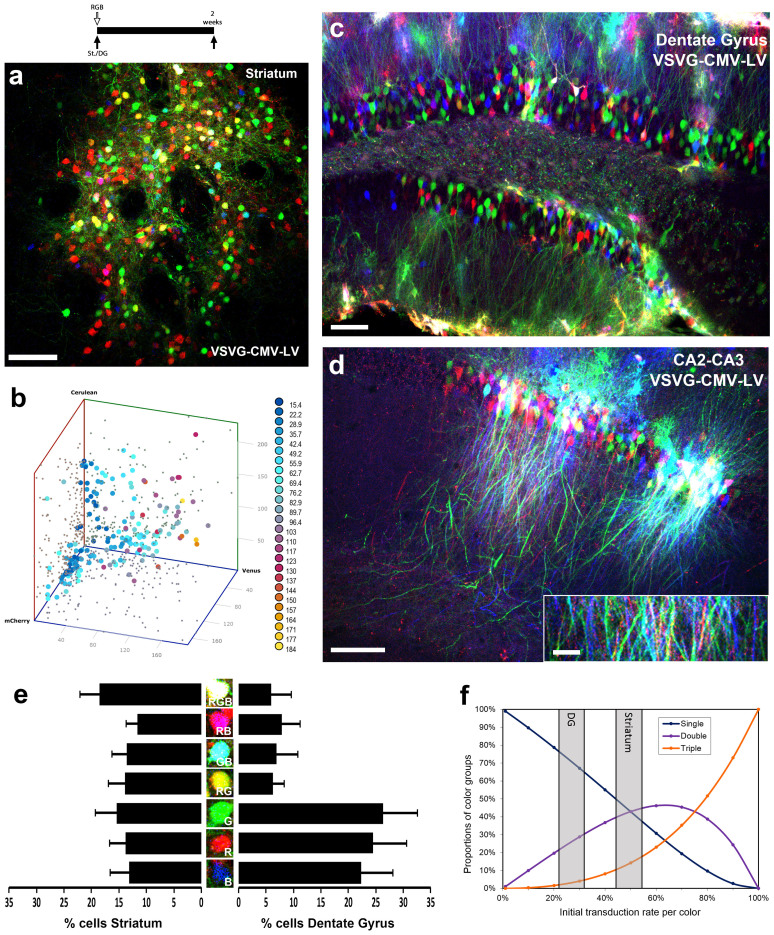
RGB multicolour marking of mature neurons. RGB marking of striatal (A), granular (C) or pyramidal (D) neurons after intraparenchymal administration of VSVG-CMV lentiviral vectors, analysed 2 weeks after injection (see experimental scheme, top). (B) 3D representation of the RGB brightness of single cells detected in (A). Fluorescence from mCherry (X-axis), Venus (Y-axis) or Cerulean (Z-axis) was quantified separately and plotted in a 3D scatter. Total RGB perceived brightness, calculated as (0.299xR) + (0.587xG) + (0.114xB) is color-coded and shown on the right hand side of (B). (E) Detail of individual neuronal bodies reproducing some examples of RGB marking, single-labelling (R, G or B), double-labelling (RG, GB or RB) or triple-labelling (RGB) cells, together with the average distribution of colour combinations (%cells) in the striatum or dentate gyrus, shown as mean ± SEM. (F) Overlay of the distribution of colour combinations found in DG and Striatum as analysed in (E) and the mathematical model^12^ of RGB marking in dependence of the initial transduction rate per vector (excluding non-transduced cells). Low transduction rates predominantly result in single-positive cells (red or green or blue cells only, shown by the blue curve in the graph). With higher transduction rates double-positive cells (purple curve) as well as triple-positive cells (orange curve) appear. Ultimately, at 100% transduction rate for each of the three vectors, all cells will be triple-positive. (A, C, D, E) Fluorescence is shown in red (mCherry), green (Venus) and blue (Cerulean). All images were analysed using confocal microscopy. Scale bar: A, 100 µm; C, D, D (insert), 25 µm.

The generation of VSVG-pseudotyped lentiviral vectors with the CMV promoter (VSVG-CMV-LV) allows for the preferential targeting of cells from the neuronal lineage^20^. Therefore, we assayed the RGB multicolour marking technique *in vivo*, at three brain sites enriched in post-mitotic neurons (striatum, dentate gyrus and CA2-CA3 layer; Figure 2). Two weeks after the stereotaxic administration of the VSVG-CMV-LV in the striatum we could observe the combinatorial expression of the three fluorescent proteins (mCherry, Venus and Cerulean; Figure 2a), reproducing the RGB principle previously observed *in vitro* (Figure 1). The applied vector dose allows for the identification of single cells, each one individually marked by a specific hue resulting from a defined combination of the three basic colours (Figure 2b), as shown by data arising from the quantification of the fluorescence of the three channels in the 179 distinguishable neurons presented in Figure 2a. Similarly, RGB marking at the dentate gyrus of the hippocampus led to the labelling and filling of the soma and dendritic tree of individually colour-coded mature neurons (Figure 2c). A similar picture was seen when labelling CA2-CA3 neurons, where we could observe combinatorial RGB marking of soma, dendrites and axonal projections (Figure 2d, 2d insert). The distribution of colour combination (% of single, double or triple-labelled cells; Figure 2e) was in excellent agreement with theoretically expected values^11^,^12^ (Figure 2f), but also resembled the data obtained *in vitro* in this work (Supplementary Figure 2) and previously reported^12^. Only some differences in the distribution of the colour groups was noted depending on the area analysed, highlighting tissue-specific factors defining the transduction efficiency (Figure 2f).

### The differential use of promoters in RGB vectors allows the tracing of cell lineages originated from the subventricular zone, evidencing a robust gliogenic activity

**Figure 3:**
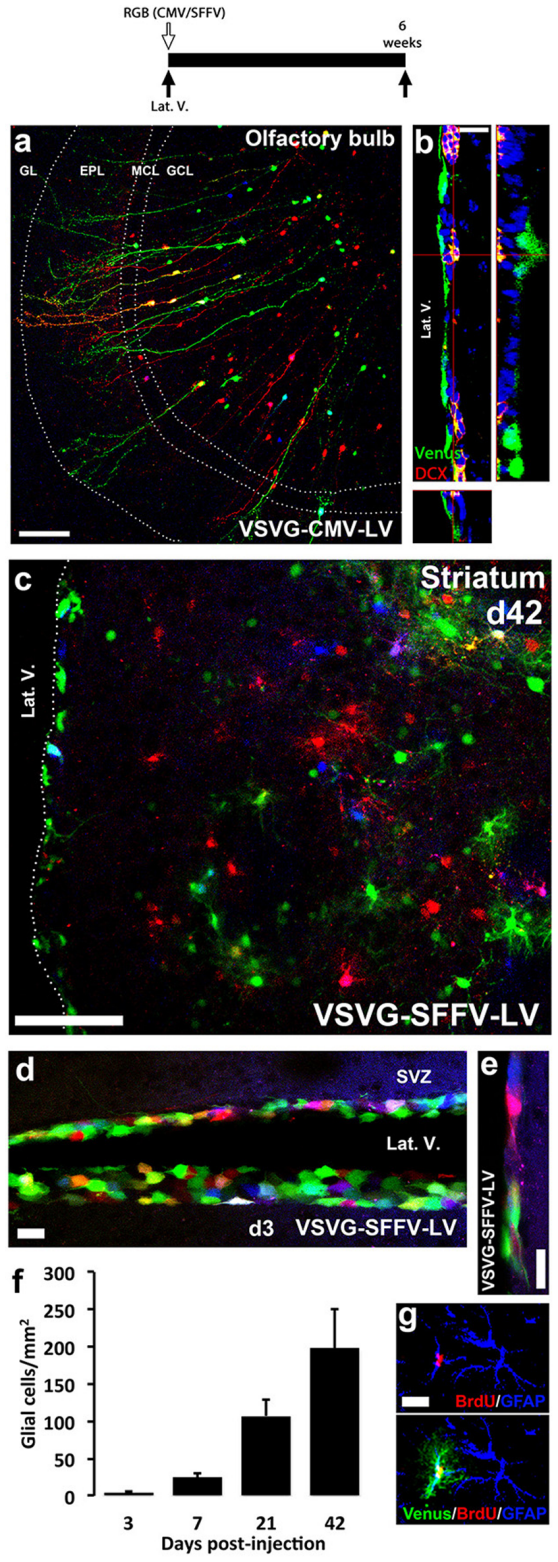
RGB tracing of cell generation from the SVZ. RGB marking by the intraventricular administration of VSVG-CMV (A, B) or VSVG-SFFV (C, D–G) lentiviral vectors (see experimental scheme, top). (A) RGB marked new neurons in the olfactory bulb, 6 weeks after intraventricular delivery of VSVG-CMV lentiviral vectors. (B) Detailed view of the SVZ, showing confocal lateral projections (right, bottom) of Venus-expressing newly generated neuronal precursors (DCX+, red), in the SVZ, 6 weeks after intraventricular delivery of VSVG-CMV (Venus) lentiviral vector. (C) RGB-marked new astrocytes in the striatum, 6 weeks after intraventricular delivery of VSVG-SFFV lentiviral vectors. (D) RGB marking in the lateral ventricles, 3 days after intraventricular delivery of VSVG-SFFV lentiviral vectors. (E) Detail of RGB marking of migratory neuroblasts, 3 days after intraventricular delivery of VSVG-SFFV lentiviral vectors. (F) Time-course analysis of newly generated glial cells in the striatum, represented as mean ± SEM cells/mm^2^. (G) Incorporation of BrdU (red) in newly generated astrocytes (GFAP+, blue), detected 21 days after intraventricular delivery of Venus expressing VSVG-SFFV-LV (green). (A, C, D–E) Fluorescence is shown in red (mCherry), green (Venus) and blue (Cerulean). All images were analysed using confocal microscopy. Lat. V., lateral ventricle; GL, glomerular layer; EPL, external plexiform layer; MCL, molecular cell layer; GCL, granular cell layer. Scale bar: A, 100 µm; B, 20 µm; C, 100 µm; D, E, G, 25 µm.

Given the *in-vivo* potential of RGB tracing of brain populations (Figure 2) we explored the application of the vectors to trace the production, migration and differentiation of new cells from the subventricular zone neurogenic niche (Figure 3). The intraventricular administration of RGB mixtures of VSVG-CMV-LV vectors labelled cells generated at the subventricular zone, and we could visualise them after migration and differentiation into new neurons in the olfactory bulb, six weeks later (Figure 3a). The RGB-labelled, newly generated neurons displayed normal migratory and morphological patterns, with their soma being preferentially located at the granular cell layer (GCL), extending the apical dendritic tree towards the external plexiform layer (EPL) (Figure 3a). As described above for the labelling of mature neurons, newly generated neurons displayed the features of the RGB-marking principle, allowing the colour identification of single-, double- and triple-labelled cells. Moreover, we could see that there was stable expression of the fluorescent proteins in neural stem cells, and their ability to provide new neural precursor cells was apparently not altered, as identified by Venus+ DCX+ cells at the subventricular zone, six weeks after injection (Figure 3b).

In contrast, we found that the administration of VSVG-SFFV-LV (SFFV instead of CMV promoter) in the lateral ventricle did not effectively provide RGB labelling of mature neurons, probably due to low expression strength in those cells (no RGB-labelled cells were found in the olfactory bulb; data not shown). However, VSVG-SFFV-LV vectors did label glial cells generated from the subventricular zone, analysed six weeks after the injection (Figure 3c). Intrastriatal injection of VSVG-SFFV-LV did not result in labelling of resident glial cells, supporting a gliogenic origin of the glial cells shown in Figure 3c. Newly generated astrocytes of many different colours migrated towards the striatum parenchyma, depicting the colour mixing principle of RGB marking for single-cell analyses (Figure 3c). A time-course analysis of this phenomenon showed that only SVZ cells are labelled 3 days after intraventricular injection of VSVG-SFFV-LV (Figure 3d), some cells showing the migratory morphology characteristic of neuroblasts (Figure 3e). With time, we could observe a progressive increase in the number of VSVG-SFFV-LV positive glial cells in the striatum (Figure 3f), identified as newly generated astrocytes (GFAP+) by their incorporation of BrdU (Figure 3g).

The present results illustrate the potential of the multicolour RGB-tracing technique to target the generation, migration and differentiation of new cells from the subventricular neurogenic niche, following the neurogenic or gliogenic pathways, by using the CMV or the SFFV promoters respectively.

### RGB γ-retroviral vectors facilitate tracing of newborn granule cells of the dentate gyrus at the single-cell level

**Figure 4:**
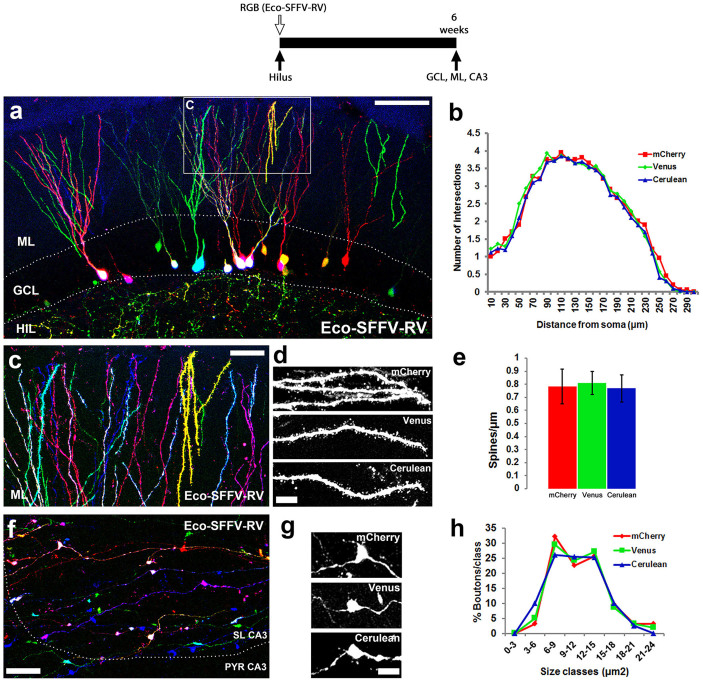
RGB tracing of hippocampal neurogenesis. RGB marking by the intraparenchymal (hilus) administration of ecotropic SFFV γ-retroviral (Eco-SFFV-RV) vectors (see experimental scheme, top). (A) RGB-marked new neurons in the dentate gyrus, 6 weeks after the intraparenchymal delivery of Eco-SFFV-RV. (B) Sholl analysis of the complexity of the dendritic arborisation of newly generated neurons, represented as mean number of intersections at a given distance from soma (µm). (C) Detail of the distal segments of the dendrites of RGB-marked new neurons (in A). High-power magnification of single-marked dendritic distal segments (D, black and white), used to analyse dendritic spine density (E; spines/µm). (F) Detail of the synaptic boutons of RGB-marked new granule neurons, projecting to the CA3 layer. High-power magnification of single-marked synaptic boutons (G, black and white), used to analyse bouton complexity and maturation, as %boutons/size class (H). (A–H) Fluorescence is shown in red (mCherry), green (Venus) and blue (Cerulean). All images were analysed using confocal microscopy. ML, molecular layer; GCL, granule cell layer; HIL, hilus; SL CA3, stratum lucidum CA3; PYR CA3, stratum pyramidale CA3. Scale bar: A, 100 µm; C, F, 20 µm; D, G, 5 µm.

We next aimed at targeting the neural stem cell pool in the subgranular layer of the dentate gyrus (DG), in order to achieve RGB tracing of newly generated granule cells (Figure 4). We used a γ-retroviral approach, to specifically target the dividing cell pool and label proliferating neural stem cells without marking mature granule cells. A first approach using VSVG-SFFV-RV did not deliver optimal transduction rates of the neurogenic pool at the DG, therefore reducing the generation of mixed colours and also not offering optimal expression strength to analyse the cells by fluorescence. We optimised the vectors by using the mouse ecotropic envelope (Eco-SFFV-RV), previously described to be effective in the transduction of neural stem cells^6^. Eco-SFFV-RV vectors facilitated effective and reproducible RGB labelling of newly generated granule cells, six weeks after the injection (Figure 4a). We could identify individually labelled granule cells, easily distinguishable based on their distinct colours, as illustrated by different 3D rendering methods (Supplementary Movie 1). Expression of the three fluorescent proteins (mCherry, Venus, Cerulean) did not influence the differentiation of new granule cells as evidenced by their morphological similarity: there are no statistically significant differences in the Sholl analysis (Figure 4b). The combinatorial expression of the three fluorescent proteins also permitted RGB marking of the dendritic compartment in the molecular layer (ML; Figure 4c) and the dendritic spines (Figure 4d) whose density was not influenced by the expression of the different fluorescent proteins, showing non-significant statistical differences (Figure 4e). Similarly, RGB marking of newly generated granule cells permitted the identification of their axonal compartment (mossy fibers), projecting to the CA3 area of the hippocampus (Figure 4f). Tracing of dentate neurogenesis with Eco-SFFV-RV allowed the identification of RGB-coded synaptic boutons at the CA3 layer, which showed no morphological differences related to the expression of the three fluorescent tags (Figure 4h).

**Figure 5:**
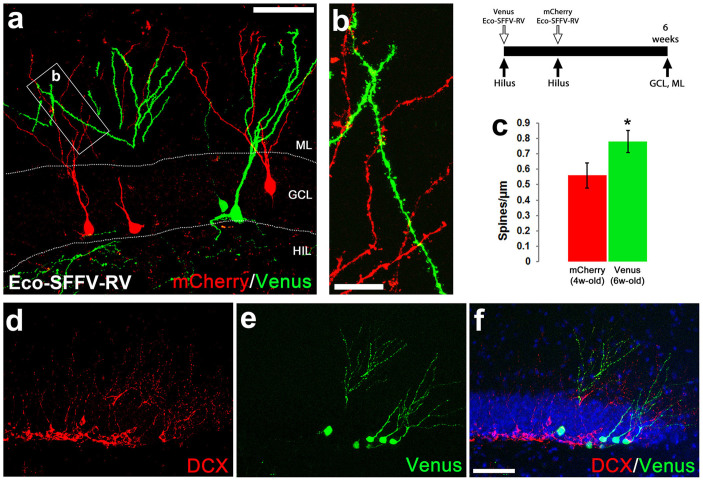
Timing of hippocampal neurogenesis with γ-retroviral vectors. Sequential marking of neuron generation in the dentate gyrus by the intraparenchymal (hilus) administration of ecotropic SFFV γ-retroviral (Eco-SFFV-RV) vectors (see experimental scheme, top right). (A) Venus+ (6 weeks-old) or mCherry+ (4-weeks-old) marked new neurons in the dentate gyrus, after the sequential delivery of Eco-SFFV-RV. (B) Detail of the distal segments of the dendrites of Venus+ or mCherry+ new neurons (in A), used to analyse dendritic spine density (C; spines/µm). (D–F) Expression of DCX (red, D, F) in neuronal precursors, next to Venus-expressing newly generated neurons (E, F; 6 weeks-old), in the dentate gyrus. All images were analysed using confocal microscopy. ML, molecular layer; GCL, granule cell layer; HIL, hilus. Statistical differences: *p<0.05. Data were analysed with a t-test. Scale bar: A, 100 µm; B, 20 µm; (D–F), 100 µm (in F).

To further extend the use of the RGB vectors, we applied the γ-retroviral vectors to analyse the temporal dynamics of neurogenesis in the dentate gyrus (Figure 5). We performed sequential tracing in the dentate gyrus with Venus Eco-SFFV-RV (green) and mCherry Eco-SFFV-RV (red), spaced by a two-week delay (Figure 5; scheme). γ-retrovirally-marked new neurons were analysed four weeks after the second (mCherry) labelling, thus differentiating between 6-weeks old (Venus+) and 4-weeks old neurons (mCherry+)(Figure 5a). The continuing maturation between 4 and 6 weeks was identified by the time-dependent increase in the dendritic spines density (Figure 5b, 5c). The use of the RGB γ-retroviral vectors was shown to be compatible with the immunohistochemical detection of markers of the neurogenic sequence, evidenced by the detection of DCX+ in neural progenitors in Venus-traced 4 weeks-old neurons (Figure 5f). To summarise, these results validate the application of RGB marking for the labelling and dynamics of newly generated cells at the dentate gyrus, together with the study of their migration, differentiation and maturation.

## Discussion

The interest of the neuroscience community in techniques that enable the analysis of cellular populations at the single-cell level and to follow their differentiation and function has attracted a significant effort in recent years. These efforts crystallized in the generation of Brainbow mice, an elegant and useful method allowing the transgenic combinatorial expression of fluorescent proteins creating multiple colours^1^. The Brainbow approach inspired the creation of several transgenic methods and their application to the study of the brain complexity^21^. More recently, novel methods based on the *in-utero* delivery of plasmids encoding fluorescent proteins, namely the StarTrack^8^, MAGIC^9^ and CLoNe^10^ methods, have allowed for the developmental tracking of cell populations, providing valuable insights to the study of brain development.

The present results are the first evidence of retro-/lentiviral-vector-mediated multicolour single-cell tracking in the CNS, complementing transgenic- and plasmid-based approaches. Our data define an experimental toolbox with a large number of potential applications for the study of the development, physiology and pathology of the brain (summarised in Supplementary Table 1). We used the RGB-marking technique with LeGO vectors for labelling and tracing of neurons, astrocytes and newly generated cells from the neurogenic niches, as well as for experiments to follow neuronal maturation. Potential future applications of these techniques are various and powerful, ranging from the study of brain anatomy and connectivity to the gene modification of multiple genes in different cell populations in a single animal. For example, various combinations of colour-encoding vectors could be delivered at different regions of the CNS, to further analyse cellular connectivity or migration. A similar approach could be applied by delivering RGB combinations at discrete stages of brain development or pathology, to further perform an end-point multi-analysis. For example, in this study we have shown that RGB marking allows for the identification of mature neurons at the single-cell level, as well as the identification of its dendritic and axonal projections, facilitating tract tracing. This particular feature could, however, be further improved by adding a palmitoylation sequence tethered to the fluorescent proteins, which allows for a preferential labelling of axonal processes vs. the somato-dendritic compartment^1^. Other combinatorial marking strategies based on trans-synaptic viral tools have been successfully used to the analysis of brain connectivity^22^,^23^,^24^. In this direction, we predict that the combination of pseudorabies virus with RGB marking and LeGO vectors will allow the exploration of uncharted connectivity and functions of the brain.

In this work, using the sequential delivery of two vectors we have shown, for example, that neuronal maturation in the dentate gyrus follows a defined temporal pattern, complementing previous work in the field^6^. Moreover, and as described in the Results, the selective use of different promoters or envelopes could help to analyse neuronal and glial cell populations at a set time. By selecting a specific envelope protein to produce viral pseudotypes, targeting of viral vectors can be modified. The presence of the particular receptor is a *conditio sine qua non* for attachment and membrane fusion leading to the transduction of a given cell. Especially if different cell types are present, many more criteria influence transduction efficiencies like receptor density, cell-cycle status, accessibility, or expression of anti-viral restriction factors. Nevertheless, the observed characteristics of RGB marking *in vivo* in the brain were in agreement with the previously proposed mathematical model^12^ balancing single colour vs multicolour labelling. Keeping the injected volume constant, decreasing vector dose (titre or volume) favours single-colour marking with lower overall fluorescence intensity, while high titres or lower tissue diffusion favour multicolour combinations as shown here (Figure 2). When multicolour labelling is achieved, the observed rates of the different colour combinations resemble the predicted ones^12^, with differences depending on the transduction rates obtained in the different brain regions (Figure 2e,f), highlighting that the comparison of *in vitro* and *in vivo* transduction rules is accurate. The promoter choice influences the cell marking on a very different level, the level of transcription. Although the vector is already integrated into the host cell's genome, this does not necessarily lead to a high expression level of the fluorescent protein encoded by that vector. Depending on the transcription factors available in a given cell type, the enhancer/promoter might be very active or almost quiescent. The specificity of cell-type specific promoters relies on this principle. Even though SFFV and CMV are ubiquitous promoters, there are important differences in their transcriptional activity. Here we took advantage of these properties to analyse glia-cell generation from the SVZ, providing novel views about the degree of renewal or colonisation of striatal areas, underestimated by previous studies^25^. We found that the SFFV promoter is selectively used by glial cells derived from the SVZ, whereas the CMV promoter more specifically expressed in newly generated neurons.

The combinatorial application of RGB marking with three colours, the use of γ-retroviral or lentiviral vectors, and the use of different promoters and envelopes make a very diverse number of applications feasible. An additional on/off system (Cre/lox) is provided in the LeGO vectors, together with drug-resistance modules^13^, expanding even further the range of potential uses. The packaging capacity of the vectors is large enough to express additional genes or markers like a Luciferase in conjunction with the fluorescent proteins for RGB marking (unpublished data), allowing whole-body bioluminescence imaging of *ex-vivo* transduced and injected stem or tumour cells. Moreover, RGB marking in the brain can be implemented in a comparatively short time frame of a few weeks, facilitating its translation to other laboratories in the field of neuroscience (for details see Supplementary Table 2).

In the study of neurogenesis the use of RGB marking with LeGO vectors has turned out to be very powerful. Analysis of the generation, migration and differentiation of newly generated cells in the developing and adult brain has historically used a combination of several experimental approaches, ranging from the use of thymidine analogues, the detection of stage- or cell-specific markers, the use of γ-retroviral tracing or the analysis of transgenic mice^26^. We here describe a toolbox with potential to complement and simplify some of these methods, providing a universal approach to study neurogenesis. The set of vectors we have described permits the spatial and temporal tracing of neurogenesis, and the customisable overexpression or downregulation of multiple different genes (a minimum of one shRNA and one cDNA per vector)^13^.

The use of multicolour coding for the study of the CNS is rapidly changing, as several laboratories are currently developing independent approaches to tackle common technical problems. Recently, the Brainbow transgenes were adapted to an AAV-vector based approach, providing a method to induce non-inheritable colour coding of CNS populations^7^. Our current approach is a complement to the available tools, providing a toolbox of viral vectors with inheritable colour coding that can be applied to the study of quiescent neurons and glia as well, but, in addition, it can also be used to track the fate of proliferating cells. A recent report by Garcia-Marques *et al*. provides an elegant approach to perform clonal analysis of astrocyte development, by the stochastic combination of six fluorescent proteins with nuclear or cytoplasmic localization, based on electroporation of plasmid and transposase in embryos^8^. Similar approaches have been recently developed by independent laboratories to study brain development^9^,^10^. These approaches have proven to be very valuable to the study of clonality and the generation and differentiation of large cell populations within the brain. However, the delivery method used by those techniques, electroporation of plasmid constructs, limits their application to the study of the embryonic^27^ or early postnatal^28^ brain. Our marking strategy and vector set are applicable to any stage of brain development, even at multiple time points, facilitating diverse experimental set-ups. RGB marking can be applied to the adult healthy brain or combined with disease models without the need of transgenic backgrounds, as recently shown by our group for the study of adult neurogenesis in a model of chronic neurodegeneration^29^.

It is important to note that there are potential limitations where both the plasmid^8^,^9^,^10^ and retroviral-vector based techniques do not fully match the experimental aims, namely clonal cell tracking. Plasmid/transposase-based approaches are effective at identifying cell populations (astrocytes, neurons) derived from a common progenitor, but unfortunately fail to isolate the identity or the location of this progenitor cell. In this work, we have been able to demonstrate single-cell analysis of mature neurons, by the use of RGB marking with lentiviral vectors (Figure 2). Importantly, application of the RGB-marking principle is more than just adding a third colour to the already previously described successful marking approaches with fluorescent proteins. In fact, generation of a large number of combinations of colours (Sup. Figure 3) increases resolution power of microscopic analyses of brain structures down to the level of individual cells and their processes, not possible with other existing techniques. The application of RGB marking for single-cell analysis could provide an optimal complement for the study of the synaptic interactions of sister or neighbouring cells in cortical columns^30^, as the combination of RGB marking with patch-clamp analysis has been recently applied with success^29^.

The idea that a combination of three (RGB marking) or several colour tags can provide precursor cells with a unique colour watermark that will remain unchanged during cell development is not yet proven for the study of the CNS. While clonal tracking has been shown to be effective for haematopoietic stem cells and terminally differentiated cells, such as hepatocytes, for tumour cells, or in cultured cell lines^11^,^31^,^32^, during brain development cells acquire different identities, taking on completely different functions in the adult brain, characterised by changes in gene activity and expression. This differentiation process could alter the levels of expression of the individual colour tags, potentially resulting in a colour shift from the originally transduced cell to its progeny in some of the clones. The combination of RGB marking with lentiviral barcoding^33^ opens the possibility to assess the clonal identity of single cells on the molecular level, allowing us to prove or disprove the feasibility of full clonal tracking by our RGB-marking technique in near future. Novel computational methods specifically developed for RGB-marked cells will help to assign single cells to a cell clone based on its colour hue in an automated way^31^. Despite the current limitations of our technical approach, RGB marking may become a useful method to study clonality (Supplementary Figure 3), as supported by our present results studying glial vs. neuronal genesis. Future experiments combining RGB marking with barcoding will allow a precise definition of the potential of this technique to analyse clonality.

To conclude, we have described a new set of experimental tools with significant potential for the study of brain function, through diverse applications of the RGB-marking technology with LeGO vectors. We propose that the method of multicolour single-cell marking with LeGO vectors will become a widely available and applied toolbox for high-resolution tracking and manipulating single neurons and glia of the CNS in health and disease.

## Methods

### Cloning of viral vectors

Three groups of viral vectors have been used in this study (Supplementary Figure 1), each group comprising of three single vectors, each of the three vectors expressing a different fluorescent protein (red, green or blue). The first group consists of LeGO vectors, which are third-generation self-inactivating HIV-1 derived lentiviral vectors. The details of LeGO vectors have already been published^14^, they are available through Addgene.org: LeGO-Cer2 (Cerulean^34^, blue, Addgene #27338), LeGO-C2 (mCherry^35^, red, Addgene #27339), LeGO-V2 (Venus^36^, green, Addgene #27340). The LeGO vectors express their fluorescent protein under the control of an enhancer/promoter of the spleen focus-forming virus^15^ (SFFV promoter). Vector maps and sequence data for all three vectors are available at http://www.LentiGO-Vectors.de. The second group of viral vectors was derived from the LeGO vectors by replacing the internal SFFV promoter with the human immediate early promoter of cytomegalovirus (CMV promoter). The CMV promoter has been PCR amplified (Fw-Primer: 5′-TATAGCGGCCGCGCTAGCTAGTTATTAATAGTAATCAATTACGGGGTC; Rv-Primer: 5′-TATAGGATCCGCGGATCTGACGGTTCACTA) from pLL3.7 (Addgene #11795) and cloned into all three LeGO vectors (red, green and blue) using NotI and BamHI. The third group of viral vectors was derived from the γ-retroviral vector RSF91.GFP.pre*^18^, whose marker gene (eGFP) has been replaced by the three fluorescent proteins Cerulean, Venus and mCherry respectively. mCherry was amplified from LeGO-C2 by PCR (Fw-Primer: 5′-ATATACCGGTCGCCACCATGGTGAGCAAGG; Rv-Primer: 5′-CGAAGTTATTAGGTCCCTCGACG) and cloned into RSF91.GFP.pre* using AgeI and BsrGI. Cerulean and Venus have been cut out from LeGO-Cer2 and LeGO-V2 and cloned into RSF91.GFP.pre* using NcoI and BsrGI. Standard procedures of molecular cloning were used to generate all viral vectors. The sequence of every DNA fragment cloned by PCR has been verified by sequencing.

### Generation of viral particles

Cell-free supernatants containing viral particles were produced by transient transfection of HEK-293T packaging cells as described^12^,^14^,^18^. In brief, lentiviral vectors were packaged using the third-generation packaging plasmids pMDLg/pRRE and pRSV-Rev^37^ as well as phCMV-VSV-G^38^ expressing the envelope protein of vesicular stomatitis virus. γ-retroviral vectors have been packaged using pcDNA3.MLVgp^16^ and phCMV-VSV-G or K73-Eco expressing the envelope protein of ecotropic murine leukaemia virus^39^. Both, supernatants containing γ-retroviral or lentiviral particles were centrifuged at 1000 × g and 4°C for 5 minutes to remove cell debris and filtered through 0.45-µm syringe filters (FP 30/0.45 CA-S, Whatman, Dassel, Germany). They have been concentrated about 100-fold by centrifugation at 8,000 × g and 6°C for 8 to 12 hours in 30 ml glass tubes using a RC-5C plus centrifuge (Sorvall, Thermo Electron, Langenselbold, Germany) with a HB-6 swinging-bucket rotor (Sorvall). After centrifugation, most of the liquid was aspirated leaving about 300 µl in the tube to resuspend the viral particles. Transferred to a microcentrifuge tube residual debris was removed by centrifuging at 8000 × g at room temperature for 1 minute. The concentrated supernatant was aliquoted to about 20 µl per tube in 200 µl tubes and frozen at −80°C.

### Titration of viral particles

Concentrated supernatants were titrated^12^ on suitable target cells, 293T cells for VSV-G pseudotypes or NIH-3T3 cells for Eco pseudotypes. For titration, target cells were incubated at 5 × 10^4^ cells in 0.5 ml medium in each well of a 24 well plate in the presence of 8 μg/ml polybrene. Viral particle containing concentrated supernatant was added to the cells as a 10-fold dilution series in PBS resulting in a final amount of 0.0001 µl to 0.1 µl of supernatant per well. The plate has been centrifuged at 1,000 × g for 1 hour at 25°C. Initial gene transfer rates were analysed 48 to 72 hours after transduction by FACS. The following cytometers were used to acquire FACS data (Becton Dickinson, Heidelberg, Germany): FACSCantoII (405/488/635 nm lasers) and LSRFortessa (405/488/561/640 nm lasers). Titres of about 1.5 × 10^9^ viral particles per ml concentrated supernatant were obtained for all vector types (range: 0.9 to 2.6 × 10^9^/ml).

### RGB transduction of cell lines

Cell culture and viral transduction were performed as previously described in detail^12^. Briefly, we transduced HEK-293T cells with the three RGB vectors at rates to ensure transduction of reasonable proportions of cells by one, two or all three vectors. For this purpose, we seeded 5 × 10^4^ 293T cells in 0.5 ml medium per well in 24-well plates. We added vector-containing supernatants, where mCherry, Venus or Cerulean vectors were prepared in equimolar amounts, to provide different multiplicities of infection (MOI), as previously described^12^. We analysed transduction by fluorescent microscopy after at least 3 days, with an Olympus IX81 Live Cell Imaging System.

### Stereotactic injection of RGB vectors

Female C57BL/6J mice (Harlan, Bicester, U.K.) were bred and maintained in local facilities. Mice were housed in groups of 4 to 10, under a 12-h light/12-h dark cycle at 21°C, with food and water ad libitum. To deliver the different combinations of RGB vectors, mice were anaesthetized with a ketamine/xylazine mixture (85 and 13 mg/kg), and 1 μl (10^9^ particles/ml; equal particle number/vector; red:green:blue = 1:1:1) of the mixture of viral particles were injected stereotaxically and bilaterally at each of the following coordinates from bregma: dentate gyrus (hilus), anteroposterior, −2.0 mm; lateral, ±1.3 mm; depth, −2 mm and anteroposterior, −1.5 mm; lateral, ±0.8 mm; depth, −2 mm; CA2-CA3, anteroposterior, −1.7 mm; lateral, ±2.2 mm; depth, −1.5 mm; lateral ventricle, anteroposterior, −0 mm; lateral, ±1 mm; depth, −2.2 mm; striatum, anteroposterior, +0.5 mm; lateral, ±2 mm; depth, −3.2 mm. All procedures were performed in accordance with U.K. Home Office licensing.

### Histological analysis of RGB-traced cells

Coronal sections were cut with a vibrating microtome from paraformaldehyde-fixed brains (50 µm). Mice perfusion, tissue processing, histological analysis and immunohistochemical detection of DCX (Santa Cruz Biotechnologies) or GFAP (Merck Millipore) expression was performed as previously described^40^. After repeated rinses with PBS, free-floating sections were mounted on to glass slides and coversliped with a Mowiol/DABCO (Sigma-Aldrich) mixture. Pulse-chase experiments with BrdU were completed as previously described^41^,^42^, administering BrdU 7 days before sacrifice (at 21 days post-tracing with viral vectors). The sections were visualised on a Leica TCS-SP5 confocal system, coupled to a Leica CTR6500 microscope. Confocal stacks were acquired using 1 µm Z steps to cover the whole morphology of the labelled cells or their processes. When required, 3D stacks were analysed using Volocity (Perkin Elmer), as shown in Sup. Movie 1.

Additionally, neurons labelled with RGB lentiviral vectors were used to determine the potential of the technique to perform single-cells studies. Briefly, using 3D confocal stacks of RGB-labelled neurons, we quantified the intensity of fluorescence in the 3 individual colour channels (red, green and blue) of each individual and distinguishable neuronal body (not overlapping with another body and not overlapping with fibre tracts). Data were represented in a 3D scatter-plot (Figure 2b), colour-coding the different points (neurons) based on the total RGB perceived brightness using the RGB to grayscale equation, in which colours are weighted according to their black-white intensity, and calculated as (0.299xR)+(0.587xG)+(0.114xB). To calculate the distribution of colour combinations (% of single, double or triple labelled cells), RGB black-white intensity was used, setting an arbitrary threshold of 40 as non-specific autofluorescence (Figure 2a).

### Characterisation of γ-retrovirally labelled dentate cells

Sholl analysis was performed on 3D reconstructions of confocal Z-stacks to analyse the dendritic tree complexity of newborn neurons^43^. Briefly, the number of crossings for concentric circles of given radii centred at the tree stem (Sholl number) was calculated. Starting radius and radius step size were set each at 10 μm; ending radius was set at 300 μm. A total of 56 neurons were analysed, of which: 16 were Venus+, 18 were mCherry+ and 22 were Cerulean+.

For the dendritic linear spine density, confocal stacks of γ-retrovirally-labelled apical dendritic processes of dentate granule cells extending through the molecular layer (ML) were acquired, generating maximum intensity projections along the z-axis. Dendritic linear spine density was calculated by dividing the total number of spines in a given dendrite by the length of that dendrite. A total of 85 dendrites were analysed, of which: 25 were Venus+, 31 were mCherry+ and 29 were Cerulean+.

Confocal stacks of γ-retrovirally-labelled mossy fibre axon terminals in the stratum lucidum (SL) were acquired, generating maximum intensity projections along the z-axis. Mossy fibre boutons were selected for measurement by following criteria established previously^44^. Bouton size was evaluated by manually-tracing each bouton and then measuring the enclosed area. Size groups every 3 μm^2^, starting from 0 μm^2^, were established and the percentage of boutons in each size group was quantified for each group. A total of 215 boutons were analysed, of which: 71 were Venus+, 68 were mCherry+ and 76 were Cerulean+.

All quantifications were performed with the help of the ImageJ image analysis software, additionally using NeuronJ and ShollAnalysis plug-ins.

### Statistical analysis

Data were expressed as mean ± SEM and analysed with the GraphPad Prism 5 software package (GraphPad Software). For all data sets, normality and homoscedasticity assumptions were reached, validating the application of the one-way ANOVA, followed by the Tukey post-hoc test for multiple comparisons. Differences were considered significant for p<0.05.

## Author Contributions

D.G.-N. and K.R. performed and analysed the experiments. D.G.-N., K.R., B.F. and V.H.P. designed the experiments and wrote the manuscript.

## Supplementary Material

Supplementary Information Sup Movie 1

Supplementary Information SI
