# Supplementary material for: In-vivo RGB marking and multicolour single-cell tracking in the adult brain
Source: Sci Rep. 2014 Dec 22;4:7520. doi: 10.1038/srep07520 (PMC4273606; doi:10.1038/srep07520)
Supplement: Supplementary Information — SI [file srep07520-s2.pdf]

Supplementary Information

In vivo RGB marking and multicolour single-cell tracking in the adult brain

Diego Gomez-Nicola, Kristoffer Riecken, Boris Fehse, V. Hugh Perry

Supplementary Figure 1

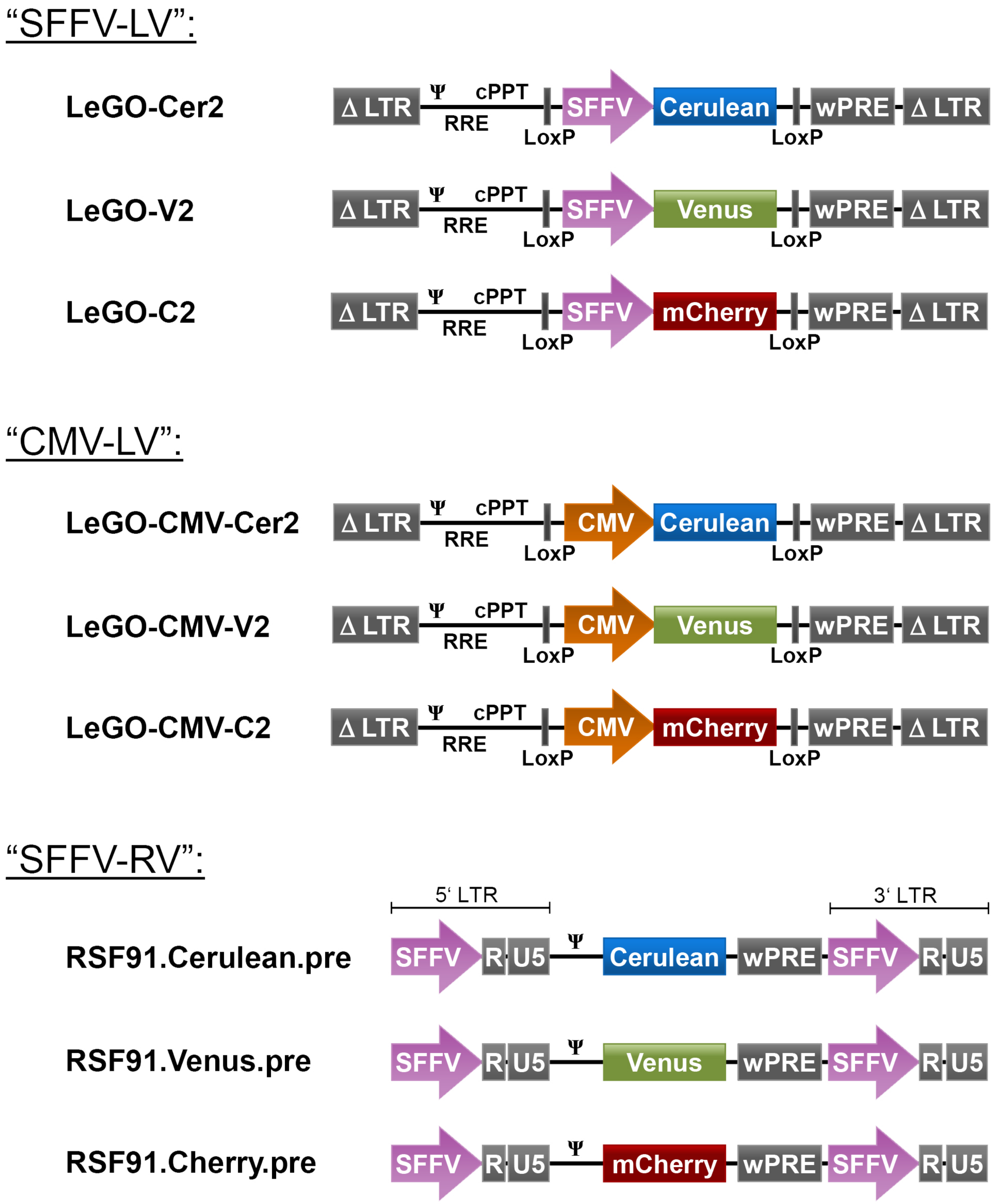

Supplementary Figure 2

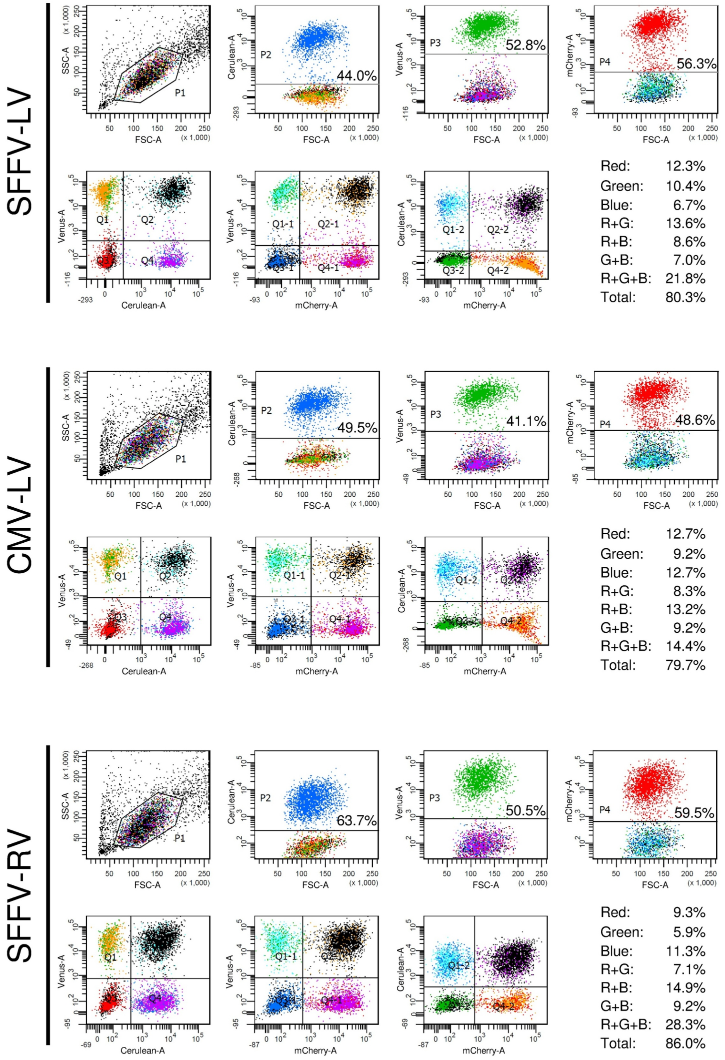

Supplementary Figure 3: Where do all the colours come from?

1.) A cell could get hit from any of the three vectors randomly:

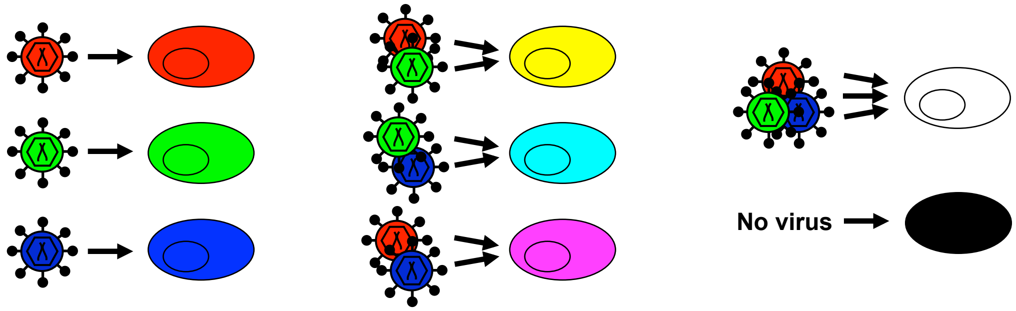

2.) Each integration site shows a different expression strength:

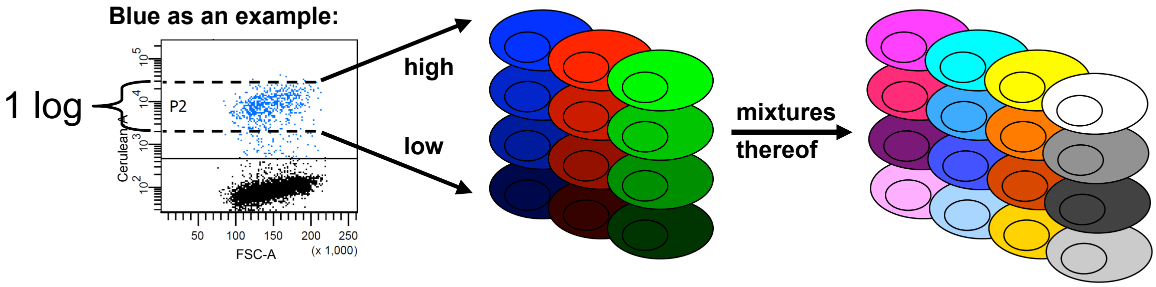

3.) Multiple vector hits lead to additional variation in expression:

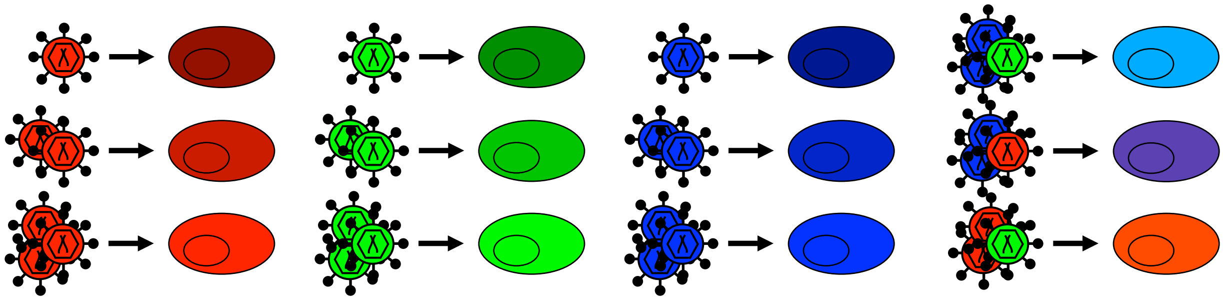

4.) How many colours do we have in total?

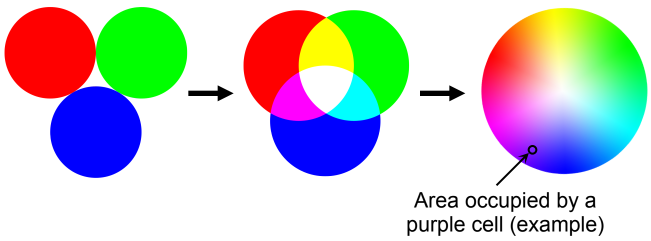

Mixing of the three basic colours in infinitesimal small increments results in an unlimited number of colours. That said, the number of colours is only restricted by the number of cells that get marked.

But: Each cell occupies a given area of the colour spectrum, thus limiting the number of colours that can be distinguished by a given observation method (exemplified by the small circle, left).

Supplementary Table 1, related to discussion. Summary of vector types and their potential applications for *in vivo* RGB marking and tracking of cells in the adult mouse brain.

Vector-Type	Transducing preferentially	Envelope	Possible Applications
CMV-LV	Neurons	VSV-G	• Marking and tracking of adult neurons (quiescent or not).<br>• Marking and tracking of neuronal progenitors migrating from subventricular zone to olfactory bulb.<br>* Simultaneous tracing and mapping of the neuronal connectivity of multiple independent brain regions<br>* Genetic reprogramming of adult neurons
SFFV-LV	Glia cells	VSV-G	• Marking and tracking of gliogenesis from the subventricular zone.<br>* Colony tracking analysis of prenatal gliogenesis
SFFV-RV	Proliferating stem cells	Eco	• Marking of the proliferating cells within the neural stem cell pool at the dentate gyrus neurogenic niche and tracing of newly generated granule cells.<br>• Analysis of temporal dynamics of neurogenesis in the dentate gyrus.<br>* Gene up-/down-regulation of multiple genes, colour encoded, modifying cell fate.<br>* Multicolour phenotyping of the different cells of the neurogenic niche

• application presented in this study
* supposed application

Supplementary Table 2, related to discussion. Timing of RGB marking experiments *in vivo*.

Step	Timing	Procedure
Production of viral particles	3 days	Transfection of producer cells and harvest of supernatant
Concentration of viral particles	1 day	Centrifugation
Titration of viral particles	3 days	Transduction of cell line and FACS analysis to calculate titer
Injection of mice	1 day	Injection of viral particles into desired region(s) of mouse brain
Latency	>3 days	Wait for the cells to express the fluorescent proteins. Wait for the cells to migrate/proliferate, depends on experimental needs
Preparation of brain tissue	3 days	Preparation, fixation and sectioning of brain tissue
Image acquisition	1 day	Microscopic analysis of sections

SUPPLEMENTARY FIGURE LEGENDS

Supplementary Figure 1. Schematic representation of viral vectors, shown as integrated provirus

Three sets of viral vectors have been used in this study, each set consisting of three vectors expressing a fluorescent protein in one of the tree basic colours red, green or blue. SFFV-LV and CMV-LV are third generation self-inactivating HIV-1 derived lentiviral vectors (LeGO vectors[14] or derivatives thereof) only differing in their internal promoter (SFFV or CMV). SFFV-RV are LTR-driven γ-retroviral vectors derived from RSF91.GFP.pre*[18]. ΔLTR = self-inactivating long terminal repeat; Ψ = *Psi*, packaging signal; RRE = Rev response element; cPPT = central polypurine tract; LoxP = recognition site of cre recombinase; SFFV = promoter of spleen focus-forming virus; CMV = immediate early promoter of human cytomegalovirus; Cerulean, Venus, mCherry = fluorescent protein cDNA; wPRE = Woodchuck hepatitis virus posttranscriptional regulatory element; R, U5 = elements of LTR.

Supplementary Figure 2. FACS analyses of new vector sets

FACS analyses of the three vector sets tested in vitro in 293T cells (compare supplementary Figure 1). The initial transduction rate per vector is given within the FACS-Plots for the red, green and blue vector respectively (please note there is significant overlap between these populations). The table shows the resulting sizes of all colour groups, e.g. Red indicates cells expressing the red fluorescent protein only, R+G indicates cells expressing the red and the green fluorescent protein, R+G+B indicates cells expressing all three fluorescent proteins (there is no overlap between these groups). Total indicates the total transduction rate of the cells, which is identical to the sum of the given seven colour groups. Analyses have been made on a BD LSRFortessa flow cytometer using three lasers (405nm, 488nm, 561nm) at 5 to 6 days after transduction with the given vector sets.

Supplementary Figure 3. Where do all the colours come from?

Schematic representation of the different determinants of the colour identity of cells traced with RGB marking.

Supplementary Movie 1

Animated sequence of a 3D rendered confocal stack from a dentate gyrus traced with an RGB combination of Eco-SFFV-RV vectors (see Figure 4A). Z stack was obtained using 1µm steps with a Leica SP5 confocal system. 3D stack was rendered using Volocity (Perkin Elmer), and is shown as a sequence of fluorescence view, followed by a close-up view of the fluorescence in the RGB channels, followed by a maximum projection of fluorescence, followed by an isosurface view of the cell somas, ending with a field view in fluorescence.
